# A Bayesian natural cubic B-spline varying coefficient method for non-ignorable dropout

**DOI:** 10.1186/s12874-020-01135-3

**Published:** 2020-10-07

**Authors:** Camille M. Moore, Samantha MaWhinney, Nichole E. Carlson, Sarah Kreidler

**Affiliations:** 1 Center for Genes, Environment and Health, National Jewish Health, 1400 Jackson St., Denver, CO, 80206 USA; 2 Department of Biostatistics and Informatics, Colorado School of Public Health, University of Colorado Denver, 17th Pl., Mail Stop B119, Aurora, CO, 80045 USA; 3 Sunrun Inc., 717 17th St., Suite 200, Denver, CO, 80202 USA

**Keywords:** Reversible jump Markov chain Monte Carlo, Missing data, Dropout, Varying coefficient model, HIV

## Abstract

**Background:**

Dropout is a common problem in longitudinal clinical trials and cohort studies, and is of particular concern when dropout occurs for reasons that may be related to the outcome of interest. This paper reviews common parametric models to account for dropout and introduces a Bayesian semi-parametric varying coefficient model for exponential family longitudinal data with non-ignorable dropout.

**Methods:**

To demonstrate these methods, we present results from a simulation study and estimate the impact of drug use on longitudinal CD4 ^+^ T cell count and viral load suppression in the Women’s Interagency HIV Study. Sensitivity analyses are performed to consider the impact of model assumptions on inference. We compare results between our semi-parametric method and parametric models to account for dropout, including the conditional linear model and a parametric frailty model. We also compare results to analyses that fail to account for dropout.

**Results:**

In simulation studies, we show that semi-parametric methods reduce bias and mean squared error when parametric model assumptions are violated. In analyses of the Women’s Interagency HIV Study data, we find important differences in estimates of changes in CD4 ^+^ T cell count over time in untreated subjects that report drug use between different models used to account for dropout. We find steeper declines over time using our semi-parametric model, which makes fewer assumptions, compared to parametric models. Failing to account for dropout or to meet parametric assumptions of models to account for dropout could lead to underestimation of the impact of hard drug use on CD4 ^+^ cell count decline in untreated subjects. In analyses of subjects that initiated highly active anti-retroviral treatment, we find that the estimated probability of viral load suppression is lower in models that account for dropout.

**Conclusions:**

Non-ignorable dropout is an important consideration when analyzing data from longitudinal clinical trials and cohort studies. While methods that account for non-ignorable dropout must make some unavoidable assumptions that cannot be verified from the observed data, many methods make additional parametric assumptions. If these assumptions are not met, inferences can be biased, making more flexible methods with minimal assumptions important.

## Background

Dropout is a common problem in longitudinal clinical trials and cohort studies, and is of particular concern when dropout occurs for reasons that may be related to the outcome of interest. For example, HIV studies are often longitudinal in nature, and it is well documented that many subjects have missing observations due to death or disease progression, leading to concerns of non-ignorable dropout [1]. Dropout is not ignorable when missingness depends on the values of the unobserved outcomes, even after conditioning on the available data [2]. In this scenario, standard longitudinal data analyses can produce biased results.

This work was motivated by the challenges associated with comparing laboratory markers of HIV disease progression and treatment response between drug users and other subjects in the Women’s Interagency HIV Study (WIHS). Illicit and recreational drug use has been hypothesized to accelerate HIV disease progression by directly enhancing virus replication and by impairing immune responses. While laboratory in vitro and animal studies suggest that drug and alcohol use impairs immune function and increases HIV replication, results from epidemiological studies have been mixed[3]. These conflicting results may be in part linked to differential dropout between drug users and other subjects. Similar dropout related challenges have been identified in quality of life data from clinical trials of cancer therapies [4], anti-depressant clinical trials,[5] and studies of smoking cessation programs [6], among others. Considering the potential impact of non-ignorable dropout on the results of statistical analyses is particularly important in this context.

While all methods that account for non-ignorable dropout rely on unavoidable assumptions that cannot be verified from the observed data [7], many methods make additional parametric assumptions about the distribution of dropout times or the functional form of the relationship between regression coefficients and dropout time. This paper reviews common parametric methods to account for non-ignorable dropout and introduces a Bayesian semi-parametric varying coefficient generalized linear mixed model to more flexibly accommodate dropout. This method extends existing frequentist natural cubic B-spline varying coefficient methods to account for dropout in longitudinal studies with a Gaussian outcome[3, 8] to other non-normal outcomes in the exponential family. Fitting the model in a Bayesian framework allows the number and location of spline knots to be jointly modeled with other model parameters, removing dependence on the choice of knots and more accurately characterizing model uncertainty. We illustrate how inference differs between parametric and semi-parametric models to account for dropout in the analysis of longitudinal changes in CD4 ^+^ T cell count and viral load suppression in the WIHS.

### Background on the WIHS

The WIHS is an ongoing prospective study of the natural and treated histories of HIV infection in women, with behavioral data and specimens collected at semiannual visits by multiple sites since 1994 [9]. In contrast to male populations, HIV and AIDS are more prevalent among women of color exposed through heterosexual partners or intravenous drug use [10, 11]. Two common measures of disease progression measured in the WIHS are CD4 ^+^ T cell count, a measure of immunologic health, and viral load, a measure of the concentration of HIV-1 RNA in the blood. For HIV ^+^ subjects that have initiated highly active antiretroviral therapy (HAART), the primary measure of treatment effectiveness is suppression of viral load (HIV-1 RNA below detection limits). The goal of our analyses is to understand the impact of drug use on disease progression and treatment response. In untreated subjects, we compare longitudinal changes in CD4 ^+^ T cell count and viral load suppression between subjects that report hard drug use and other subjects in the WIHS, as there is evidence to suggest that hard drug use in particular can dampen immune response and increase virus replication. Rates of treatment initiation among hard drug users are lower or treatment occurs later for a variety of reasons, including provider perceptions that they are unable to keep appointments, are not ready for treatment, have unstable living situations, are unable to fill prescriptions or have limited ability to adhere to treatment. In addition, non-physician providers, are more likely to care for illicit drug users and to resist prescribing HAART [12]. Thus, the number of hard drug users initiating treatment is limited. In addition, any recreational drug use may potentially reduce compliance to HAART regimens. Therefore, for treated subjects in the WIHS, we compare longitudinal viral load suppression between recreational drug users and others.

**Fig. 1:**
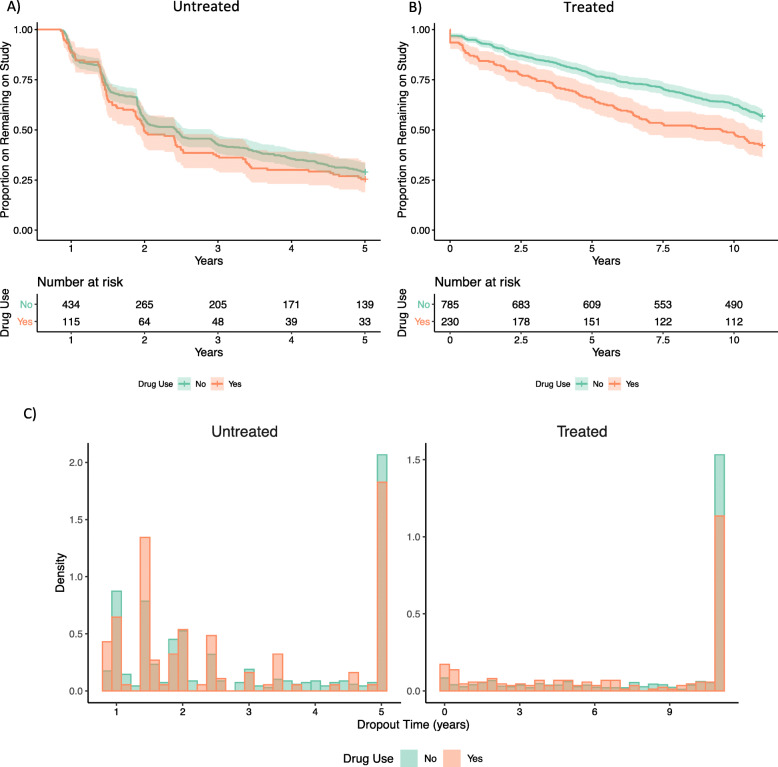
Dropout in the WIHS: Kaplan-Meier Curves for Study Dropout for (**a**) Untreated Subjects Reporting Hard Drug Use (Dashed) and Others (Solid) and (**b**) Treated Subjects Reporting Recreational Drug Use (Dashed) and Others (Solid). Histograms of dropout time are presented in panel (**c**)

**Fig. 2:**
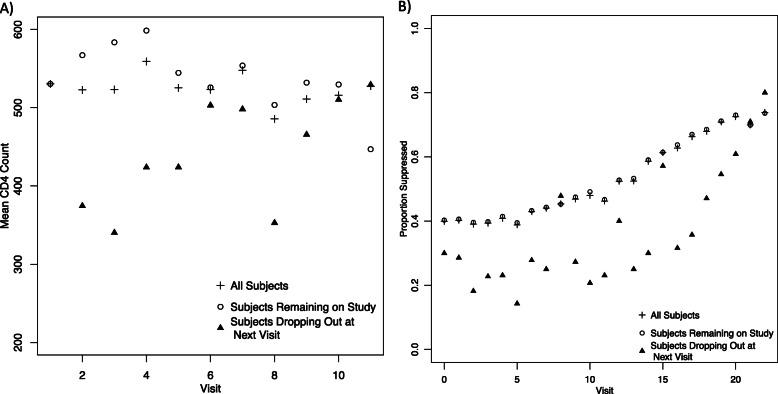
CD4 ^+^ and Viral Load Suppression Over Time in the WIHS. **a** Mean CD4 ^+^ by Visit for All Untreated Subjects (crosses), Subjects Remaining on Study to the Next Visit (open circles) and Subjects Dropping Out at the Next Visit (closed triangles) and **b** Proportion with Undetectable Viral Load for All Treated Subjects (crosses), Subjects Remaining on Study to the Next Visit (open circles) and Subjects Dropping Out at the Next Visit (closed triangles)

In our initial investigation into the data, we found several causes for concern. We noted that a large proportion of subjects dropped out of the study early, with half of untreated subjects lost by 2.4 years (median of 4 observations, Fig. 1a) and a quarter of treated subjects lost by 5 years (median of 19 observations, Fig. 1b) after treatemtn initiation. In addition, drug users tended to drop out of the study earlier than other subjects and were more likely to die within 1 year of their last study observation (Table 2). Due to the prevalence and differential distribution of dropout, missing data could have a large impact on the results of our analysis. Untreated subjects that dropped out of the study had lower mean CD4 ^+^ at their last visit compared to subjects that remained on study (Fig. 2a), and treated subjects that dropped out of the study early were less likely to have suppressed viral load (Fig. 2b). This suggests that subjects that dropped out may have done so due to more rapidly deteriorating health, raising concerns of non-ignorable dropout.

## Methods

### Background on methods to account for missing data

Dropout is not ignorable and data are missing not at random when missingness depends on the values of the unobserved outcomes, even after conditioning on the available data [2]. Selection, frailty and mixture models are likelihood based approaches that can account for data that are missing not at random. While there are several methods to account for non-ignorable dropout in longitudinal studies with a Gaussian response, methods for non-normal data are less developed [13]. The literature is particularly sparse for addressing non-ignorable dropout in GLMMs in semi-parametric or Bayesian frameworks.

#### Selection models

Selection models factor the joint distribution of the outcomes, ***y***, which include both observed and missing values, and missing data indicators, ***r***, as *f*(*y*|*x*)*f*(*r*|*y*,*x*). Frequentist parametric selection models for Gaussian outcomes have been proposed by several authors [14–16], and parametric selection models for binary outcomes have been proposed by Ibrahim et al.[17] and Wu and Wu [18]. Identification of parameters in selection models can be challenging and relies on distributional assumptions for the outcome and a parametric relationship for how potentially missing outcome data are related to the probability of missingness. In addition, selection models typically require specialized numerical routines for maximizing the likelihood, which can limit practical utility for broad ranges of problems [6].

#### Fraily models

Frailty models, also called shared parameter models, factor the joint distribution of the outcome and missing data indicators as $\int f(y | x, \eta) f(r | x, \eta)dF(\eta |x)$, where *η* are the shared parameters or frailties that induce dependence between the outcomes and missing data indicators. Parametric frailty models have been proposed for both Gaussian and non-normal outcomes [19–23]. Identification of parameters in frailty models is driven by the parametric frailty distribution. This choice is often arbitrary, and may influence the validity of results [6]. Another key assumption of frailty models is that the repeated measures are independent of drop-out times conditional on the frailties.

For example, Schluchter proposed a two stage frailty model, which we will consider in our simulation study and application. The first stage assumes that each subject’s responses follow a linear regression with random intercept *b*_*i*0_ and slope *b*_*i*1_, which can be written ***y***_*i*_=***X***_*i*_***b***_*i*_+***e***_*i*_, where ***e***_*i*_ is a vector of independent, normally distributed error terms for subject *i*. In the second stage, the subject-specific random coefficients and the natural log of dropout time, *u*_*i*_, are modeled with a joint multivariate normal distribution:

$$\left(\begin{array}{c} \boldsymbol{b}_{i}\\ \log(u_{i})\end{array}\right) \sim \mathrm{N}\left(\boldsymbol{\mu} = \left(\begin{array}{c} \boldsymbol{\mu}_{b} \\ \mu_{u} \end{array}\right),\boldsymbol{\Sigma} = \left(\begin{array}{cc} \Sigma_{b}& \sigma_{bu}' \\ \sigma_{bu}& \sigma_{u}^{2} \end{array}\right) \right) $$

where ***μ***_*b*_ is the mean of the random coefficients, *μ*_*u*_ is the mean of the natural log of dropout time, *Σ*_*b*_ is the covariance matrix of the random coefficients, *σ*_*bu*_ is a row vector containing the covariances of *u* and each random coefficient, and $\sigma _{u}^{2}$ is the variance of the natural log of the dropout times. This model allows the underlying slope and intercept to be associated with dropout time, via the covariance parameters *σ*_*bu*_. If these covariances are zero, then the random coefficients and dropout time are independent and dropout is assumed to be non-informative. In addition to assuming a log-normal distribution of dropout times, the model assumes that there is a linear relationship between the log of dropout time and the dropout time specific intercepts and slopes, since the random coefficients are related to dropout time through the covariance parameters. Violations of these assumptions can lead to biased estimates and inaccurate inference.

#### Mixture models

Mixture models factor the joint distribution of the outcome and missing data indicators as *f*(*y*|*r*,*x*)*f*(*r*|*x*) [19, 24–28]. Pattern mixture models [29] are a popular method to account for non-ignorable missingness when missingness can be categorized into distinct patterns. After classifying data according to missing data patterns, models can be fit to the outcome data within each pattern. Kaciroti et al. have described Bayesian pattern mixture models for binary and count data,[30–32] however, these methods may not be feasible for large numbers of dropout patterns or continuous dropout times. For example, in the WIHS, follow-up visits were intended to occur every 6 months, but the exact timing of visits varies greatly between subjects, so that observation times are not aligned and dropout may occur at any continuous point in time.

Varying coefficient models (VCM) are another mixture model approach that more easily accommodate continuous dropout times. VCMs adjust for dropout by allowing regression coefficients to depend on dropout time. For example, for a Gaussian distributed outcome, the response vector for subject *i* is modeled using a linear mixed model, with regression coefficients that depend on dropout time, such that ***y***_*i*_=***X***_*i*_***β***(*u*_*i*_)+***Z***_*i*_***α***_*i*_+***e***_*i*_, where ***β***(*u*_*i*_) are the dropout varying regression coefficients, ***X***_*i*_ is the design matrix for the fixed effects, **Z**_*i*_ is the design matrix associated with the random effects, ***α***_*i*_, and ***e***_*i*_ is a vector of normally distributed error terms. If the regression coefficients are constant with respect to dropout time, the model reduces to a standard generalized linear mixed model (GLMM). Assuming regression coefficients are linear (or low-order polynomial) functions of dropout time results in Wu and Bailey’s conditional linear model (CLM) [25]. However, if the regression coefficients are not linearly related to dropout time (or the polynomial function is mis-specified) estimates can be biased [8, 33]. For Gaussian outcomes, semi-parametric varying coefficient models that only require that regression coefficients are smooth, continuous functions of dropout time have been proposed, making them more robust [8, 33]. In a Bayesian framework, methods for binary outcomes have utilized marginalized transition models for population level rather than subject-specific inference.

### Bayesian varying coefficient models for non-ignorable dropout

We introduce a Bayesian natural cubic B-spline varying coefficient GLMM (BNSV) that can account for dropout in longitudinal studies with exponential family outcomes, while avoiding assumptions about the distribution of dropout times or the functional form of the relationship between regression coefficients and dropout time, common in parametric frailty and mixture models. This method extends existing frequentist natural cubic B-spline varying coefficient methods to account for dropout in longitudinal studies with a Gaussian outcome[3, 8] to other non-normal outcomes in the exponential family. Similar models have been proposed for Gaussian outcomes using penalized splines [34].

Fitting the semi-parametric varying coefficient model in a Bayesian framework has several advantages. The number and location of spline knots control the smoothness, shape, and flexibility of the spline over the range of dropout times; however, fitting in a frequentist framework, it is unclear how to choose these parameters [35–39]. We utilize a reversible jump Markov chain Monte Carlo (RJMCMC) approach that jointly models the number and location of knots for the spline and does not require the choice of a single set of spline knots to make statistical inference. In addition, there is no need to specify a parametric distribution for the dropout times or to use an extra bootstrap simulation to estimate standard errors, as is required in the frequentist, semi-parametric approach.

#### VCM for longitudinal exponential family outcomes

Let $\boldsymbol {y}=(\boldsymbol {y_{1}} \dots \boldsymbol {y}_{m})'$ be the set of outcomes observed on *m* subjects with *n*_*i*_ observations each at times $\boldsymbol {t}=(\boldsymbol {t_{1}} \dots \boldsymbol {t}_{m})'$. Let $\boldsymbol {u}=(u_{1} \dots u_{m})'$ be the set of *m* observed dropout times. First we describe the conditional model for ***y***|***u***, which allows the change in the outcome over time to depend on dropout time and results in dropout time specific estimates.

For exponential family outcomes, the observation specific conditional VCM is:

1 $$\begin{array}{*{20}l}  f(y_{ij}|u_{i}, \boldsymbol{\alpha}_{i},\eta_{ij}) &= \exp \left[ \{y_{ij}\eta_{ij}-b(\eta_{ij})\}/\phi \right] c(y_{ij}, \phi)  \\ \mu_{ij}=E(y_{ij}|\eta_{ij}) &=b'(\eta_{ij}) \\ g(\mu_{ij}) = \eta_{ij} &= \beta_{0}+ \beta_{1}(u_{i})t_{ij} + \boldsymbol{C}_{ij}\boldsymbol{\beta}_{C}+ \mathbf{Z}_{ij} \boldsymbol{\alpha}_{i} \end{array} $$

where *g*() is the link function, *η*_*ij*_ is the linear predictor, **Z**_*ij*_ is the design matrix associated with the random effects, ***α***_*i*_, and *ϕ* is a scale parameter. For a model with a random intercept and slope, let $\boldsymbol {\alpha _{i}} = \left [\begin {array}{ll} \alpha _{0i} \\ \alpha _{1i} \end {array}\right ] \sim N\left (\left [\begin {array}{ll} 0 \\ 0 \end {array}\right ], \left [\begin {array}{ll} \sigma _{{0}}^{2} & \sigma _{01} \\ \sigma _{01} & \sigma _{{1}}^{2} \end {array}\right ]\right)$. *β*_0_ is the intercept, and *β*_1_(*u*_*i*_) is the dropout-varying slope. ***C***_*ij*_ is the design matrix associated with the covariate effects, ***β***_***C***_, which do not depend on dropout time.

#### Natural cubic b-splines

The slope, *β*_1_(*u*_*i*_), in Eq. 1 is assumed to be a smooth function of dropout time and is modeled using natural cubic B-splines [40]. The ith subject’s dropout-time specific slope is $\beta _{1}(u_{i}) = \sum _{k=1}^{D+1} \theta _{k} \tilde {B}(\boldsymbol {u}, D, \boldsymbol {l})_{[i,k]}$. Here *D* is the number of degrees of freedom for the dropout-varying component of the slope and $\tilde {B}(\boldsymbol {u}, D, \boldsymbol {l})$ is the matrix of natural cubic B-spline basis functions evaluated at ***u*** with *D*+1 knots (including 2 boundary knots) at locations ***l***={*l*_1_,...,*l*_*D*+1_}, for *D*≥1. For *D*=0, there is no dropout-varying effect and $\tilde {B}(\boldsymbol {u}, D, \boldsymbol {l})_{[i,1]} = 1$ for all subjects. $\boldsymbol {\theta }=(\theta _{1}\dots \theta _{D+1})$ are the coefficients associated with the basis functions.

#### Dropout time model and Bayesian bootstrapping

While inference conditional on ***u*** can be made without assumptions about the distribution of ***u***, it is often of interest to summarize the results with a marginal or “dropout adjusted” estimate of the outcome that does not depend on dropout time, which requires integrating over the distribution of dropout times. We utilize Rubin’s Bayesian bootstrap method [41] to flexibly model the distribution of dropout times, to estimate the proportion of subjects dropping out at each observed dropout time, and to calculate marginal estimates in a straightforward manner [34].

The Bayesian bootstrap repeatedly samples the proportion of subjects dropping out at each of the observed dropout times, rather than re-sampling the observed dropout times themselves, as would be done in a frequentist bootstrap. Define $\boldsymbol {u^{0}} = (u^{0}_{1},..., u^{0}_{R})$ as the *R* unique ordered observed dropout times. Let $\boldsymbol {\pi }=(\pi _{1},\dots, \pi _{R})$ be the vector of probabilities of dropping out at each observed dropout time and $\boldsymbol {N}=(N_{1},\dots, N_{R})$ be the number of subjects observed dropping out at each unique dropout time. The likelihood is proportional to $\prod _{r=1}^{R} \pi _{r}^{N_{r}}$. If we assume the prior distribution of ***π*** is proportional to $\prod _{r=1}^{R} \pi _{r}^{-1}$, the posterior distribution of ***π*** is proportional to $\prod _{r=1}^{R} \pi _{r}^{N_{r}-1}$, which is the kernel of a Dirichlet distribution. The posterior distribution of ***π*** is then Dirichlet with concentration parameters ($N_{1},\dots,N_{R}$).

#### Calculation of marginal effects

Working on the linear predictor scale, it is possible to calculate a marginal slope, averaged over both the distribution of dropout times and random effects. Note that the calculation of the marginal slope depends on the assumption that subjects continue on the same trajectory after their dropout. The expected value of the linear predictor at time t is:

$$\begin{aligned} E(\eta_{ij}|t, C) &=\int \int \left\{\beta_{0} + \beta_{1}(u)t + \boldsymbol{C}\boldsymbol{\beta}_{C}+ \mathbf{Z} \boldsymbol{\alpha} \right\} dF(\boldsymbol{\alpha}) dF(u|C) \\ &=\int \left\{\beta_{0} + \beta_{1}(u)t + \boldsymbol{C}\boldsymbol{\beta}_{C} \right\} dF(u|C)\\ &=\beta_{0} + \boldsymbol{C}\boldsymbol{\beta}_{C} +t \int \beta_{1}(u)dF(u|C) \end{aligned} $$

*E*(*η*_*ij*_|*t*,***C***) is also a linear function of time with slope *β*1′=*E*[*β*_1_(*u*)|*C*]. If we assume the distribution of dropout times does not depend on the covariates (*F*(*u*|***C***)=*F*(*u*)), then *β*1′=*E*[*β*_1_(*u*)], and the marginal slope can be estimated at each iteration of the RJMCMC algorithm in a straightforward manner. At iteration *s*, ${\beta }_{1}^{'(s)}=(\boldsymbol {\pi }^{(s)})^{T} {\beta }_{1}(\boldsymbol {u^{0}})^{(s)}$.

If the assumption that the distribution of dropout times does not depend on the covariates is inappropriate, it may not always be possible to easily estimate marginal slopes, particularly in more complex cases where the distribution of dropout times may depend on continuous covariates or several different covariates. However in simple cases, for example comparing the change in the outcome over time between treatment or drug use groups, marginal effects can be easily calculated, even if the distribution of dropout times depends on group. Here, the Bayesian bootstrap can be performed separately for each group and group specific marginal slopes can be calculated, as shown in our application to the WIHS.

#### Prior distributions

*D* is assumed to have a Poisson(*λ*) prior distribution [35]. For knot locations, we assume a discrete set of *M* candidates, such as the order statistics of the observed drop out times. For a given *D*, all sets of knots are assumed to have the same prior probability, so that $p(l_{1}...l_{D+1}|D) = {M \choose D+1}^{-1} = \frac {(D+1)!(M-D-1)!}{M!}$.

The fixed effect coefficients for the natural B spline basis functions are assumed to have a multivariate normal prior with mean zero, and independent covariance structure, such that ***θ***∼*MVN*_*D*+1_(0,***R***_0_), where $\boldsymbol {R}_{0} = \sigma _{\beta }^{2} I_{D+1 \text { x} D+1}$. *I*_(*D*+1)x(*D*+1)_ is a (*D*+1) x(*D*+1) identity matrix. In practice, $\sigma _{\beta }^{2}$ is chosen to be large enough to be “non-informative.” We similarly assume (*β*_0_,***β***_***C***_)^′^ have a multivariate normal prior, $MVN(0, \sigma _{\beta }^{2} I)$. In addition, we assume an inverse Wishart prior for the covariance of the random effects, and for a normally distributed outcome, an inverse gamma prior for the variance of the residual error.

#### Estimation and implememtation

A RJMCMC algorithm [42] is used to fit the BNSV model and has been implememted in the InformativeDropout R package available at https://github.com/kreidles/informativeDropout. Full details of the sampler and a discussion of implementation issues can be found in Section 1 of the supplementary material.

## Simulation study

### Methods compared and data simulation

We assess the performance of the BNSV, CLM,[25] and Schluchter’s parametric frailty model[21] in estimation of the marginal slope (expected change in the outcome over time) as well as dropout time specific slopes. We chose to compare to the CLM and parametric frailty models as these are popular methods that are straight-forward to implement.

Simulated data were generated under four different scenarios, including two normally distributed outcomes and two binary outcomes. In these four scenarios the slopes were related to dropout time by two different dropout mechanisms: (i) a continuous and smooth function meeting assumptions of the BNSV and (ii) a discontinuous step function. In addition, simulations for linear dropout-varying slopes and no dropout effect are presented in Section 2 of the supplementary material (available online) and illustrate that the BNSV can also fit CLMs and GLMMs.

**Fig. 3:**
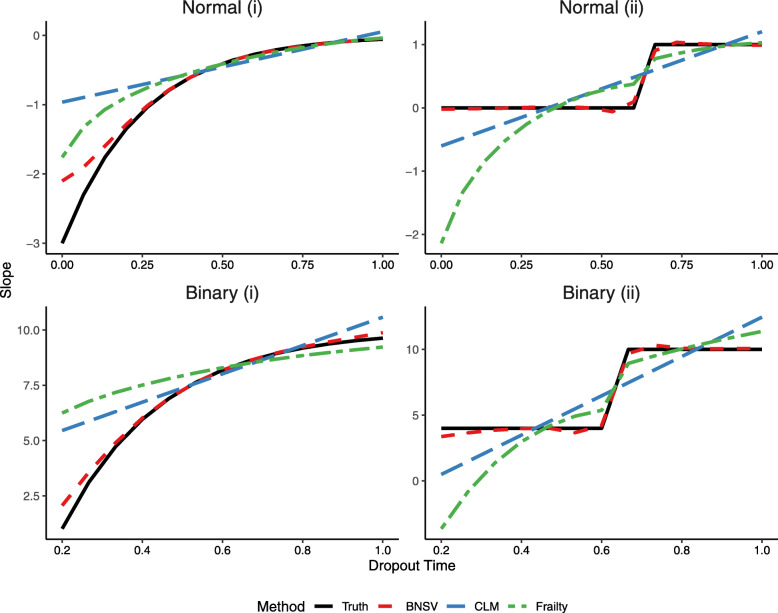
Simulation Study: Comparison of the Posterior Mean Estimates of the Dropout-Varying Slope for the BNSV, CLM, and Frailty Models

More specifically, the following form for the data was assumed: *η*_*ij*_=*β*_0_+*β*_1_(*u*_*i*_)*t*_*ij*_+*α*_0*i*_+*α*_1*i*_*t*_*ij*_,*i*=1...*m*,*j*=1...*n*_*i*_ for *m* subjects with *n*_*i*_ observations for the ith subject, where (*α*_0*i*_,*α*_1*i*_)^′^∼*N*(0,***Σ***_*α*_). For the Gaussian simulations, $y_{ij}|\eta _{ij} \sim N(\eta _{ij}, \sigma _{\epsilon }^{2})$, and *β*_0_=0. Dropout times were *u*=*U*/15∈[0,1], resulting in 16 time points spaced equally from 0 to 1. Uniform dropout was created from a beta-binomial where *p*∼*Beta*(1.5,1.5) and *U*∼*Bin*(15,*p*). The within-subject variance, $\sigma _{\epsilon }^{2}$, was set at 0.067. The elements of ***Σ***_*α*_ were as follows: $\sigma _{0}^{2}=0.4, \sigma _{1}^{2}=0.01$ and *σ*_01_=−0.01. These simulation settings were developed in other papers that tested methods for analyzing non-ignorable dropout in a frequentist setting. [8, 33] For the binary simulations, *y*_*ij*_|*η*_*ij*_∼*Bernoulli*(logit^−1^(*η*_*ij*_)),*β*_0_=−3, and for stability, dropout began at the third observation. The elements of ***Σ***_*α*_ were as follows: $\sigma _{0}^{2}=0.4, \sigma _{1}^{2}=0.1$ and *σ*_01_=−0.01. The forms of the dropout-varying slope were: Normal (i): *β*_1_(*u*)=−3 exp(−4*u*), Normal (ii): *β*_1_(*u*)=*I*_(*u*>2/3)_, Binary (i): *β*_1_(*u*)=10{1−2 exp(−4*u*)}, Binary (ii): *β*_1_(*u*)=4+61_(*u*>2/3)_ (Fig. 3). The magnitude of the dropout effects in these scenarios were similar to those seen in the WIHS and other typical HIV cohort studies. For each simulation scenario, 1000 datasets with 400 subjects each were created.

### Methods of evaluation

The BNSV and Bayesian versions of the CLM and frailty models were fit to each dataset, as well as a naive GLMM that did not account for dropout. The performance of the methods was evaluated graphically and in terms of bias, variance, and mean square error for the marginal slope, estimated by the posterior mean. All analyses were implemented in R using custom MCMC algorithms utilizing the splines, MASS, mvtnorm, MCMCpack, and gtools packages. An R package to implement BNSV models is available at https://github.com/kreidles/informativeDropout.

### Implementation

For the BNSV, a maximum of 10 degrees of freedom were considered for the dropout-varying component of the slope, for a maximum of 11 total degrees of freedom for the slope. The prior mean for the number of degrees of freedom for the dropout-varying component of the slope was set to 5, and the prior variance for the coefficients was set to 25 for the normal simulations, and 100 for the binary simulations. The prior for *Σ*_*α*_ was *IW*(3,*I*) and the prior for $\frac {1}{\sigma _{\epsilon }^{2}}$ was *IG*(0.001,0.001). The probability of proposing a birth/dimension increase was 0.2. The MCMC chain was initiated with five equally spaced knots (including the 2 boundary knots) and coefficients set to their least squares or WLS estimates. Random effects were started at 0. Chains were run for 40,000 iterations with a burn in of 10,000 without thinning.

### Results

Model performance was quantified in terms of bias, variance, and mean squared error (MSE) for the marginal slope (Table 1). The GLMM had the lowest variance, likely because the method makes unmet assumptions that simplify the model and also has the fewest parameters. The BNSV method had the lowest bias and MSE for the marginal slope in all scenarios. Graphs of the predicted BNSV, frailty model, and CLM slopes at each dropout time are presented in Fig. 3. The BNSV method was able to more accurately describe the relationship between dropout time and the slope compared to both the frailty model and the CLM, which always fits a linear relationship. While in some cases the CLM had low bias or MSE for the marginal slope, it had poor model fit and did not perform well in the estimation of dropout time specific slopes. For example, in the Binary (ii) simulation, the CLM under-estimates slopes at early dropout times and over-estimates slopes at later dropout times (Fig. 3), such that these errors are averaged out in the marginal slope calculation, despite the poor model fit.

**Table 1:** Simulation Study Comparing BNSV, CLM, Frailty and GLMM Models: Posterior Mean Marginal Slope Estimates, Bias (Relative Bias), Variance and Mean Squared Error (MSE)

	**Normal (i)**				**Normal (ii)**			
Method	**Slope**	**Bias**	**Var.**	**MSE**	**Slope**	**Bias**	**Var.**	**MSE**
BNSV	-0.64	**0.06(9%)**	0.01	**0.02**	0.35	**-0.002 (-1%)**	0.01	0.01
CLM	-0.45	0.25 (36%)	0.003	0.06	0.30	-0.05 (-14%)	0.03	0.03
Frailty	-0.52	0.19 (27%)	0.004	0.04	0.16	-0.19 (-54%)	0.01	0.04
GLMM	-0.21	0.49 (70%)	**0.001**	0.24	0.65	0.31(89%)	**0.002**	0.10
	**Binary (i)**				**Binary (ii)**			
Method	Slope	Bias	Var.	MSE	Slope	Bias	Var.	MSE
BNSV	7.49	**0.13 (2%)**	0.19	**0.20**	6.67	**-0.05 (-1%)**	0.17	**0.18**
CLM	8.02	0.66 (9%)	0.11	0.54	6.47	-0.24 (-4%)	0.14	0.20
Frailty	8.14	0.78 (11%)	0.14	0.75	6.04	-0.96 (-14%)	0.18	1.11
GLMM	9.09	1.74 (24%)	**0.09**	3.11	8.56	1.85 (28%)	**0.14**	3.55

**Table 2:** Demographic Characteristics of Untreated Subjects with HIV Disease in the WIHS by Drug Use Group. Median (Interquartile Range) or Percent (N)

	**Untreated**		**HAART**	
	**Other**	**Drug User**	**Other**	**Drug User**
	**(*N*=566)**	**(*N*=248)**	**(*N*=785)**	**(*N*=230)**
Age (years)	35.1 (30.2-40.4)	37.8 (33.0-42.5)	38.7 (33.7-43.6)	40.5(34.9-45.7)
Baseline CD4 ^+^	499 (320-688)	417 (272-594)	271 (150-424)	256 (126-423)
Baseline log_10_(Viral Load)	4.0 (3.1-4.7)	4.3 (3.8-4.9)	4.1 (3.3-4.9)	4.3 (3.4-5.0)
Dropout Time (days)	713.5	404.5	4015.0	3485.0
	(394.2-1818.0)	(208.8-882.5)	(2136.0-4015.0)	(1112.0-4015.0)
Minority	84.1%(476)	81.0% (201)	84.3% (662)	76.1% (175)
Died within 1 year of dropout	4.2% (24)	11.3% (28)	24.2% (150)	34.8% (80)

## Analysis of the WIHS data

We applied the BNSV method to investigate the impact of drug use on longitudinal HIV outcomes in the WIHS. For untreated subjects, we hypothesized that hard drug users would have steeper declines in CD4 ^+^ T cell count compared to other untreated subjects in the cohort. For HIV+ subjects that have initiated highly active antiretroviral therapy (HAART), the primary measure of treatment effectiveness is suppression of viral load (HIV-1 RNA below detection limits). We hypothesized that recreational drug users would have slower increases in the odds of viral load suppression compared to other subjects in the cohort.

### Methods

We utilized the BNSV method to compare longitudinal changes in CD4 ^+^ count between consistent hard drug users and other untreated subjects and to compare viral load suppression between consistent recreational drug users and other treated subjects in the WIHS while accounting for dropout. Subjects were classified as consistent hard drug users if they reported injection or non-injection use of cocaine, opiate or amphetamine use at 50% or more of visits combined with use within the last year before dropout. Subjects were classified as consistent recreational drug users if they reported marijuana, or use of cocaine, opiate, amphetamine, or other drugs at 50% or more of visits combined with use within the last year before dropout. Dropout time was calculated as the day of the last visit + 1. Descriptive statistics are presented in Table 2.

Ln(CD4 ^+^) was modeled for untreated subjects from the initial WIHS cohort (first recruitment period) for the first 5 years of the study, beyond which many of the subjects had missing data. If a subject remained on study for longer than 5 years a dropout time of 5 years + 1 day (1826 days) was assigned. In addition to hard drug use, baseline ln(CD4 ^+^) and its interaction with time were included as covariates in the model. Viral load suppression was modeled for subjects that initiated treatment between 1995 and 2000 for all visits up to 11 years after initial treatment initiation, when many subjects no longer had available data. Again, if a subject remained on study for longer than 11 years a dropout time of 11 years + 1 day was assigned. Since detection limits of viral load assays changed over time, viral loads under 400 copies/mL were considered “undetectable". Baseline ln(CD4 ^+^) and log_10_(viral load) (measurements preceeding treatment initiation) and their interactions with time were included as covariates in the model.

Different dropout-varying slopes and dropout time distributions were allowed for drug users and other subjects. The RJMCMC chains were run for 200,000 iterations, with a burn in of 50,000 iterations. A Poisson prior with a mean of 5 was used for the number of knots in the model. Spline coefficients and covariates were updated in separate blocks. Normal distributions with mean 0 and variance of 100 were used as priors for the coefficients to be “non-informative.” Slopes on the linear predictor scale, averaged over dropout time, were calculated using the Bayesian bootstrap method. For comparison, CLMs and Schluchter’s frailty models, as well as GLMMs that did not account for dropout, were fit to the data using a similar MCMC estimation algorithm.

### Results

#### Longitudinal cD4 ^+^ count

**Fig. 4:**
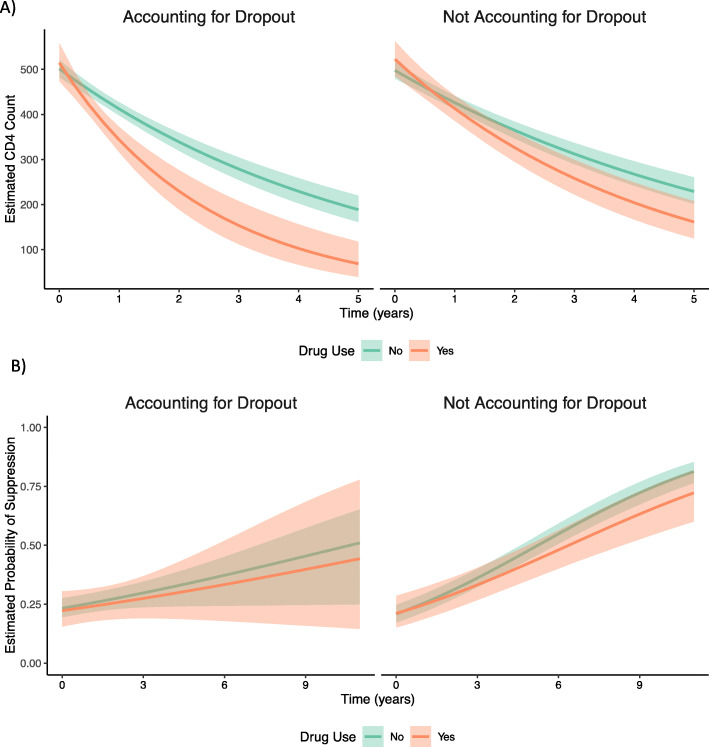
**a**) BNSV and LMM Posterior Mean CD4 ^+^ Count and 95% Credible Interval (CI) over Time in Untreated Subjects in the WIHS, Assuming a Baseline CD4 ^+^ Count of 478.5 **b**) BNSV and GLMM Posterior Mean Probability of Suppression and 95% Credible Interval (CI) over Time in a Subject that Initiated HAART in the WIHS, Assuming a Baseline CD4 ^+^ count of 267, Baseline log_10_(viral load)=4.2, and Random Effects = 0

**Fig. 5:**
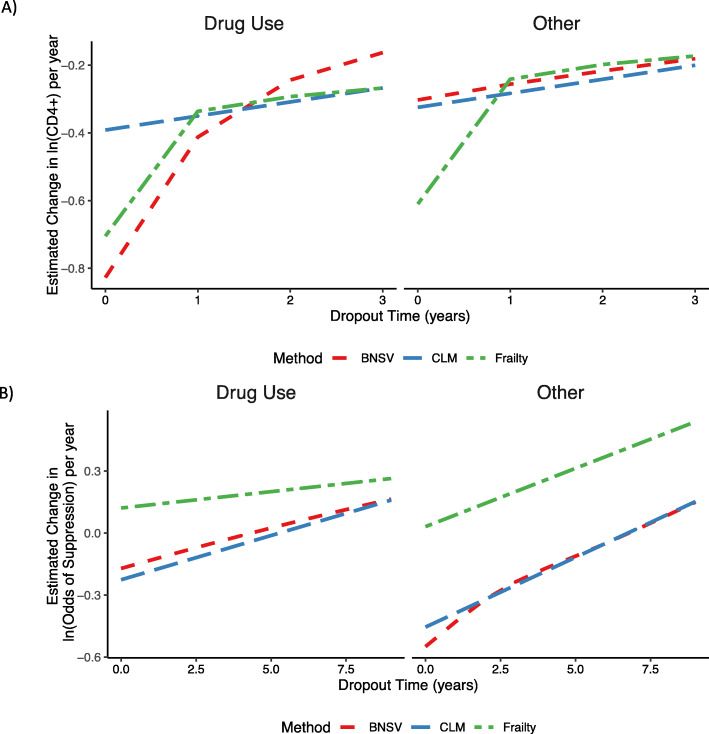
**a**) BNSV, CLM and Frailty Model Posterior Mean Estimated Changes in CD4 ^+^ Count per Year by Dropout Time for Untreated Subjects in the WIHS, Assuming a Baseline CD4 ^+^ Count of 478.5 **b**) BNSV, CLM and Frailty Model Posterior Mean Estiamted Changes in ln(Odds of Suppression) per Year for Subjects that Initiated HAART in the WIHS, Assuming a Baseline CD4 ^+^ count of 267, Baseline log_10_(viral load)=4.2, and Random Effects = 0

Consistent hard drug users tended to dropout of the study earlier and were more likely to dropout due to death (Table 2). Analyses accounting for dropout with the BNSV show that overall, hard drug users had more rapid declines in CD4 ^+^ count than those who did not use hard drugs (Fig. 4a). Assuming a baseline CD4 ^+^ count of 478.5 (median), hard drug users CD4 ^+^ counts declined by 33.5% per year (95% CI: 25.0-41.2) compared to 17.8% (95% CI: 14.9-20.7) for others in the WIHS (Table 3). Comparing these results to a linear mixed-effects model, declines in CD4 ^+^ were steeper and the magnitude of the difference between hard drug users and non-users was larger in the BNSV model (Fig. 4a). Using a linear mixed model, hard drug users were found to have 22.4% declines in CD4 ^+^ count per year (95% CI: 17.8-26.8) compared to 14.6% (95% CI: 12.1-17.0) in others. For subjects that did not report hard drug use, the changes in CD4 ^+^ count per year estimated using the CLM and frailty models were similar to the BNSV; however for subjects that did report hard drug use, the BNSV estimated larger declines in CD4 ^+^ count than the CLM or frailty model. For the CLM, this difference can be explained by the larger declines predicted by the BNSV for recreational drug users with early dropout times (Fig. 5a). For the frailty model, this is likely due to the lack of fit of the lognormal distribution for dropout times.

**Table 3:** Estimated Changes in ln(CD4 ^+^) and ln(Odds of Viral Load Suppression) per Year for Untreated Subjects in the WIHS Using the BNSV and GLMM Methods. Changes in ln(CD4 ^+^) assume a baseline CD4 ^+^ of 478.5. Changes in ln(odds) assume baseline CD4 ^+^ of 267 and baseline log_10_(viral load)=4.2. PM=posterior mean, CI=credible interval, PP=posterior probability of a difference <0, indicating steeper declines in CD4 ^+^ and less rapid increases in odds of viral load suppression among drug users

	**Others**		**Drug users**		**Difference**		
	**PM**	**95% CI**	**PM**	**95% CI**	**PM**	**95% CI**	**PP**
**A) Untreated:*Δ* ln(CD4**^**+**^**)**							
BNSV	-0.20	(-0.23, -0.16)	-0.41	(-0.53, -0.29)	-0.21	(-0.34,-0.09)	0.9996
Frailty Model	-0.19	(-0.22, -0.15)	-0.27	(-0.33, -0.22)	-0.08	(-0.15, -0.02)	0.99
CLM	-0.20	(-0.22, -0.17)	-0.27	(-0.32, -0.22)	-0.07	(-0.13, -0.02)	0.99
GLMM	-0.16	(-0.18, -0.13)	-0.24	(-0.29, -0.18)	-0.08	(-0.14, -0.02)	0.99
**B) HAART:**							
***Δ* ln(Odds of Suppression)**							
BNSV	0.11	(0.005, 0.17)	0.09	(-0.06, 0.25)	-0.02	(-0.18,0.15)	0.61
Frailty Model	0.18	(0.14, 0.23)	0.15	(0.05, 0.25)	-0.03	(-0.14, 0.07)	0.74
CLM	0.12	(0.06, 0.17)	0.08	(-0.04, 0.19)	-0.04	(-0.16, 0.09)	0.73
GLMM	0.26	(0.22,0.29)	0.21	(0.14, 0.28)	-0.05	(-0.13, 0.03)	0.90

#### Longitudinal viral load suppression

Consistent recreational drug users also tended to dropout of the study earlier and were more likely to dropout due to death than other subjects that initiated HAART (Table 2). The average change in the log odds of viral load suppression per year assuming a median baseline CD4 ^+^ count of 267 and log_10_(viral load) of 4.2 are presented in Table 3. The probability of suppression for a subject with the average slope, baseline CD4 ^+^ count of 267 and log(viral load) of 4.2 are shown in Fig. 4b. For both recreational drug users and other subjects that initiated HAART, the estimated probability of viral load suppression as well as the change in the odds of suppression over time were reduced using the BNSV method to account for dropout compared to a standard GLMM; however, estimated differences in the change in odds of suppression over time between drug users and others are similar for the two models. For a recreational drug user with baseline CD4 ^+^ count of 267, log(viral load) of 4.2, and random effects of 0, the odds of viral load suppression increased by 1.07 times per year (95% CI: 0.93 to 1.21), compared to 1.12 times per year (95% CI: 1.06 to 1.19) for a subject with the same covariates that did not use recreational drugs. Using a standard GLMM that did not account for dropout, these estimates were 1.23 (95% CI: 1.15 to 1.32) and 1.29 (95% CI: 1.24 to 1.34) respectively. While recreational drug users had smaller increases in the odds of suppression per year, this difference was not statistically significant (Table 3). The CLM showed similar results to the BNSV, likely because a linear relationship between dropout time and changes in the log(odds of suppression) fit the data well for both drug uers and others (Fig. 5b). The frailty model showed increases in the odds of suppression intermediate between the GLMM and BNSV. Again, this is likely due to lack of fit of the lognormal distribution for dropout times, which in turn influences the estimated dropout time-specific slopes via to covariance parameter, *σ*_*bu*_.

### Sensitivity analysis

The results of these analyses rely on the assumption that subjects continue on the same linear trajectory after their dropout. We test the sensitivity of our results to this assumption by considering a proportional attenuation of the slope after a subject’s drop out, such that after dropping out, a subject’s slope becomes *δβ*_1_(*u*_*i*_) (Section 3 of the supplementary material). For CD4 ^+^ declines, while the estimates of the differences between drug users and others are reduced assuming, *δ*=0.25,0.5,0.75, hard drug users still have significantly lower CD4 ^+^ counts at years 1 to 4 than other untreated subjects. For viral load suppression, the odds of suppression remain lower for consistent recreational drug users compared to other subjects that initiated HAART for *δ*=0,0.25,0.5,0.75, however, as in the primary analysis, differences between drug users and others were not statistically significant.

## Discussion

Potentially non-ignorable dropout is an important consideration when analyzing data from longitudinal clinical trials and cohort studies. While methods that account for non-ignorable dropout must make some unavoidable assumptions that cannot be verified from the observed data [7], many methods make additional parametric assumptions about the distribution of dropout times or the functional form of the relationship between regression coefficients and dropout time. If these assumptions are not met, inferences can be biased, making flexible methods with minimal assumptions important. In our simulation studies, we showed that the BNSV method, which non-parametrically models this distribution of dropout times with a Bayesian bootstrap and flexibly models the relationship between regression coefficients and dropout time with natural cubic B-splines, has reduced bias and mean squared error for the marginal slope and more accurately captures the dropout time varying slope than other methods, such as the CLM and parametric frailty models, which make additional parametric assumptions. These improvements are important since dropout time distributions and relationships between dropout time and changes in outcomes may not always follow simple parametric distributions or polynomial forms in real world analyses.

In our application to the WIHS, we find important differences in estimates of changes in CD4 ^+^ T cell count over time in untreated subjects that report hard drug use between different models used to account for dropout. We find steeper declines over time using the BNSV model, which makes fewer assumptions, compared to the CLM and frailty models. Failing to account for dropout or to meet parametric assumptions of models to account for dropout could lead to underestimation of the impact of hard drug use on CD4 ^+^ T cell count decline in untreated subjects. In our analyses of viral load suppression in subjects that intiated treatment, accounting for dropout using the BNSV showed smaller increases in viral load suppression over time compared to the frailty model and GLMM that did not account for dropout. The relationship between dropout time and the change in log(odds of suppression) was approximately linear, so that the CLM produced similar results to the BNSV. While we did not find significant differences in the odds of suppression between drug users and others in any of our analyses, the probability of suppression was lower when accounting for dropout using the BNSV or CLM. Failing to appropriately account for dropout could lead to over-estimation of the probability of viral load suppression. These low levels of suppression are concerning and require further investigation into methods to help subjects with treatment compiance and affordability of medications.

One drawback of the BNSV method is that the RJMCMC algorithm is computationally intensive; however, we did not find that computaitional times were prohibative in either our simulation study or the WIHS data analysis. For the Normal (i) and Binary (i) simulations (400 subjects, 3000-4000 observations), the BNSV took 8.8 and 19.1 minutes, respectively, to complete 40,000 iterations using a MacBook Pro with 3.5 GHz Intel Core i7 processor and 16 GB of RAM. For the WIHS analyses, the analysis of CD4 ^+^ T cell count (814 subjects, 3,196 observations) took 1.2 hours to complete 200,000 iterations; the analysis of viral load suppression took 6.2 hours to complete 200,000 iterations, due to the larger sample size (1,015 subjects / 15,909 observations) and because Metropolis Hastings steps must be used to estimate the random effects in models with a binary outcome. For comparison, the CLM took 41 minutes and 4.6 hours, and the frailty model took to 22 minutes and 3.3 hours to run the same number of iterations for the CD4 ^+^ T cell and viral load analyses respectively (Supplementary Materials, Table 5).

## Conclusions

We propose a flexible, semi-parametric natural cubic B-spline varying coefficient method to account for dropout in a Bayesian framework. The BNSV extends existing frequentist natural cubic B-spline varying coefficient methods to account for dropout in longitudinal studies with a Gaussian outcome[3, 8] to other non-normal outcomes in the exponential family, while also allowing the number and location of spline knots to be jointly modeled with other model parameters, removing dependence on the choice of spline knots and more accurately characterizing model uncertainty. The BNSV allows for dropout occurring at any continuous point in time and avoids making parametric assumptions about the distribution of dropout times or the functional form of dropout-varying slope. Results of our simulation studies show that the BNSV reduces bias and mean squared error for the marginal slope compared to parametric frailty models, CLMs and standard GLMMs when non-ignorable dropout is present. The BNSV can also accurately fit models with a linear dropout-varying effect or no dropout-varying effect.

## Supplementary information

**Additional file 1** Supplementary Material.

## Data Availability

A minimal dataset supporting the conclusions of this article and code to implement BNSV models are available as part of the InformativeDropout R package:https://github.com/kreidles/informativeDropout. Full data from the WIHS can be obtained from https://www.niaid.nih.gov/research/wihs-public-dataset.

## Abbreviations

BNSV: Bayesian natural cubic B-spline varying coefficient GLMM
CLM: Conditional linear model
GLMM: Generalized linear mixed model
HAART: Highly active antiretroviral therapy
HIV: Human immunodeficiency virus
IG: Inverse gamma
IW: Inverse Wishart
MCMC: Markov chain Monte Carlo
MSE: Mean squared error
RJMCMC: Reversible jump Markov chain Monte Carlo
VCM: Varying coefficient model
WIHS: Women’s interagency HIV study
