# Supplementary material for: A Bayesian natural cubic B-spline varying coefficient method for non-ignorable dropout
Source: BMC Med Res Methodol. 2020 Oct 7;20:250. doi: 10.1186/s12874-020-01135-3 (PMC7539484; doi:10.1186/s12874-020-01135-3)
Supplement: Supplementary file 1 — Additional file 1 Supplementary Material. [file 12874_2020_1135_MOESM1_ESM.pdf]

Supplementary Material: A Bayesian natural cubic B-spline varying coefficient method for non-ignorable dropout

Camille M. Moore, Samantha MaWhinney,
Nichole E. Carlson, and Sarah Kreidler

September 12, 2020

1 Reversible Jump MCMC Algorithm for the BNSV

Given the current set of values at iteration s of the RJMCMC algorithm, a set of values for the $s+1$ iteration is simulated as follows:

1.1 Dimension Change

At iteration $s+1$ a dimension change from $D^{(s)}$ to D^* is proposed by altering the number of spline knots included in the model. The dimension or number of knots in the model is allowed to change by 1 in either a birth or death step. Proposals of births and deaths of knots occur with probabilities b and d, respectively, with $b+d=1$, unless the minimum (or maximum) number of knots are currently in the model, in which case a birth (or death) is proposed with probability 1. First a random number, U, is drawn from a Uniform(0,1). distribution.

1.1.1 Birth Step

If $U < b$ or the minimum number of knots is in the model, a birth is proposed. In a birth step, a new knot is added to the spline for the dropout varying slope. The new knot location is chosen randomly from the discrete set of candidate knots that are not currently used in the model, with probability $\frac{1}{N-D^{(s)}-1}$. This results in a new proposed set of knots, $\boldsymbol{l}^*$.

When a knot is added to the spline, the B-spline basis functions must be recalculated based on $\boldsymbol{l}^*$ and a coefficient must be added for the additional basis function included in the spline. Our approach to adding a coefficient is based on the estimate of the coefficients from a single iteration of the WLS when $\boldsymbol{l}^{(s)}$ and $\boldsymbol{l}^*$ are used for the natural cubic B spline transformation of dropout times.

First, $\tilde{B}(\boldsymbol{u}, D^{(s)}, \boldsymbol{l}^{(s)})$ and $\tilde{B}(\boldsymbol{u}, D^{*}, \boldsymbol{l}^{*})$, the matrix of natural cubic B-spline basis functions evaluated at $\boldsymbol{u}$ with $D+1$ knots at locations $\boldsymbol{l}$ are calculated. Design matrices $\boldsymbol{X}^{(s)}$ and $\boldsymbol{X}^{*}$ for $\boldsymbol{\theta}^{(s)}$ and $\boldsymbol{\theta}^{*}$ are created as follows:

$$\boldsymbol{X} = \begin{bmatrix} \mathbf{1_1} & \boldsymbol{t_1}\tilde{B}(\boldsymbol{u}, D, \boldsymbol{l})_{[1,]} \\ \vdots & \\ \mathbf{1_i} & \boldsymbol{t_i}\tilde{B}(\boldsymbol{u}, D, \boldsymbol{l})_{[i,]} \\ \vdots & \\ \mathbf{1_m} & \boldsymbol{t_m}\tilde{B}(\boldsymbol{u}, D, \boldsymbol{l})_{[m,]} \end{bmatrix}$$

The weighted least squares (WLS) estimates of $\boldsymbol{\theta}^{(s)}$ and $\boldsymbol{\theta}^{*}$ are then calculated as follows:

1. Set $\boldsymbol{\eta}^0$ to a reasonable starting value, such as $g\left(\frac{\sum_{ij} y_{ij}}{N}\right)$. This value must be independent of the current estimates of the fixed effect spline coefficients in order for the Jacobean to be 1.
2. Calculate $\boldsymbol{\eta}^{WLS} = \boldsymbol{\eta}^0 + \boldsymbol{Z\alpha} + \boldsymbol{C\beta_C}$ and $\boldsymbol{\mu}^{WLS} = g^{-1}(\boldsymbol{\eta}^{WLS})$.
3. Calculate $\tilde{\boldsymbol{y}} = \boldsymbol{\eta}^0 + (\boldsymbol{y} - \boldsymbol{\mu}^{WLS})g'(\boldsymbol{\mu}^{WLS})$ and $\boldsymbol{W}^{-1} = b''(\boldsymbol{\eta}^{WLS})\{g'(\boldsymbol{\mu}^{WLS})\}^2$.
4. The WLS estimates for β_0 and $\boldsymbol{\theta}$ given the random effects and covariates are $(\beta_{0,WLS}, \boldsymbol{\theta_{WLS}})' = (\boldsymbol{X'WX})^{-1}\boldsymbol{X'W}\tilde{\boldsymbol{y}}$.

The knot to be added to the model l^* will lie an interval $(l_k^{(s)}, l_{k+1}^{(s)})$ for $k \in \{0, 1, \ldots, D+1\}$, so that if $k = 0$, $l^* \in (-\infty, l_1^{(s)})$ and if $k = D+1$, $l^* \in (l_{D^{(s)}+1}, \infty)$. Essentially, if the knots in $\boldsymbol{l}^*$ are put in order, k is the position of the added knot. A random number, v, is drawn from a $N(0, \sigma_v^2)$ distribution. If $k = 0$, the proposed coefficients, $(\beta_0^*, \boldsymbol{\theta}^*)'$, are then calculated as

$$\begin{bmatrix} \beta_0^* \\ \theta_1^* \\ \theta_2^* \\ \vdots \\ \theta_{D^*+1}^* \end{bmatrix} = \begin{bmatrix} \beta_{0,WLS}^* + \beta_0^{(s)} - \beta_{0,WLS}{}^{(s)} \\ \theta_{WLS,1}^* + v \\ \boldsymbol{\theta_{WLS,-1}^*} + \boldsymbol{\theta}^{(s)} - \boldsymbol{\theta_{WLS}}{}^{(s)} \end{bmatrix}$$

If $k = D^{(s)} + 1$,

$$\begin{bmatrix} \beta_0^* \\ \theta_1^* \\ \theta_2^* \\ \vdots \\ \theta_{D^*+1}^* \end{bmatrix} = \begin{bmatrix} \beta_{0,WLS}^* + \beta_0^{(s)} - \beta_{0,WLS}{}^{(s)} \\ \boldsymbol{\theta_{WLS,-D^*+1}^*} + \boldsymbol{\theta}^{(s)} - \boldsymbol{\theta_{WLS}}{}^{(s)} \\ \theta_{WLS,D^*+1}^* + v \end{bmatrix}$$

If $0 < k < D^{(s)} + 1$,

$$\begin{bmatrix} \beta_0^* \\ \theta_1^* \\ \vdots \\ \theta_{k+1}^* \\ \vdots \\ \theta_{D^*+1}^* \end{bmatrix} = \begin{bmatrix} \beta_{0,WLS}^* + \beta_0^{(s)} - \beta_{0,WLS}{}^{(s)} \\ \theta_{WLS,1}^* + \theta_1{}^{(s)} - \theta_{WLS,1}{}^{(s)} \\ \vdots \\ \theta_{WLS,k}^* + \theta_k{}^{(s)} - \theta_{WLS,k}{}^{(s)} \\ \theta_{WLS,k+1}^* + v \\ \theta_{WLS,k+2}^* + \theta_{k+1}{}^{(s)} - \theta_{WLS,k+1}{}^{(s)} \\ \vdots \\ \theta_{WLS,D^*+1}^* + \theta_{D^{(s)}+1}{}^{(s)} - \theta_{WLS,D^{(s)}+1}{}^{(s)} \end{bmatrix}$$

It can be shown that the Jacobean for this transformation is 1.

The acceptance probability for a birth step is $a(D^*, D^{(s)})$ =min(1, LAPJ), where where L is the likelihood ratio, A is the ratio of the priors, P is the ratio of proposals, and J is the Jacobian, which equals 1. For the Poisson prior for D, the uniform prior for locations given the number of knots, and the normal prior for the coefficients,
$A = \frac{\lambda}{N-D^{(s)}}(2\pi\sigma_\beta^2)^{-1/2} \exp\left[\frac{1}{2\sigma_\beta^2}\{(\beta_0, \boldsymbol{\theta})^{(s)}(\beta_0, \boldsymbol{\theta})^{(s)'} - (\beta_0, \boldsymbol{\theta})^*(\beta_0, \boldsymbol{\theta})^{*'}\}\right]$ and
$P = \frac{d(N-D^{(s)})\sqrt{2\pi\sigma_v^2}\exp(v^2/2\sigma_v^2)}{b(D^*+1)}$. Elements of the proposal and prior cancel so that $a(D^*, D^{(s)}) = \min\Big(1,$
$L\frac{d\lambda(N-D^{(s)}-1)\sigma_v}{b(D^*+1)\sigma_\beta}\exp\left[\frac{v^2}{2\sigma_v^2} + \frac{1}{2\sigma_\beta^2}\{(\beta_0, \boldsymbol{\theta})^{(s)}(\beta_0, \boldsymbol{\theta})^{(s)'} - (\beta_0, \boldsymbol{\theta})^*(\beta_0, \boldsymbol{\theta})^{*'}\}\right]\Big)$. To determine whether to accept or reject the proposal, γ is randomly sampled from a $U(0,1)$ distribution. If $a(D^*, D^{(s)}) > \gamma$, $D^{(s+1)} = D^*$, and $\beta_0^{(s+1)} = \beta_0^*$, $\boldsymbol{\theta}^{(s+1)} = \boldsymbol{\theta}^*, \boldsymbol{l}^{(s+1)} = \boldsymbol{l}^*$. Else, $D^{(s+1)} = D^{(s)}$.

1.1.2 Death Step

If $U > b$ or the maximum number of knots is in the model, then a death step is performed. In a death step, a knot is randomly selected from the $D^{(s)} + 1$ knots in $\boldsymbol{l^{(s)}}$ to remove from the model. Again, this results in a new proposed set of knots, $\boldsymbol{l}^*$ and a coefficient must be removed from the model. The death step is deterministic, so that the birth of a knot is reversible. First, calculate $\boldsymbol{\theta_{WLS}}^{(s)}$ and $\boldsymbol{\theta^*_{WLS}}$ as in a birth step.

Similar to a birth step, determine k, the position of the knot to be deleted, with $k = 1$ if the knot to delete is the first in the ordered set of $\boldsymbol{l}^{(s)}$, and $k = D^{(s)} + 1$ if it is the last. Then calculate the proposed set of coefficients,

$(\beta_0^*, \boldsymbol{\theta}^*)'$, as follows:

$$\begin{bmatrix} \beta_0^* \\ \theta_1^* \\ \theta_2^* \\ \vdots \\ \theta_{D^*+1}^* \end{bmatrix} = \begin{bmatrix} \beta_{0,WLS}^* + \beta_0^{(s)} - \beta_{0,WLS}{}^{(s)} \\ \boldsymbol{\theta_{WLS}^*} + \boldsymbol{\theta}_{[-\boldsymbol{k}]}{}^{(s)} - \boldsymbol{\theta}_{\boldsymbol{WLS},[-\boldsymbol{k}]}{}^{(s)} \end{bmatrix}$$

where the subscript $[-k]$ indicates that the kth element is deleted from the vector.

The acceptance probability for a death step is $a(D^*, D^{(s)})$ =min(1, LAPJ), where where L is the likelihood ratio, A is the ratio of the priors, P is the ratio of proposals, and J is the Jacobean, which equals 1. For the Poisson prior for D, the uniform prior for locations given the number of knots, and the normal prior for the coefficients,
$A = \frac{N-D^*}{\lambda}(2\pi\sigma_\beta^2)^{1/2} \exp\left[\frac{1}{2\sigma_\beta^2}\{(\beta_0, \boldsymbol{\theta})^{(s)}(\beta_0, \boldsymbol{\theta})^{(s)'} - (\beta_0, \boldsymbol{\theta})^*(\beta_0, \boldsymbol{\theta})^{*'}\}\right]$ and
$P = \frac{b(D^{(s)}+1)}{d(N-D^*)\sqrt{2\pi\sigma_v^2}\exp(v^2/2\sigma_v^2)}$, where $v = \theta_k^{(s)} - \theta_{WLS,k}^{(s)}$. Elements of the proposal and prior cancel so that:
$a(D^*, D^{(s)}) = \min(1,$
$L\frac{b(D^{(s)}+1)\sigma_\beta}{d\lambda(N-D^*-1)\sigma_v} \exp\left[-\frac{v^2}{2\sigma_v^2} + \frac{1}{2\sigma_\beta^2}\{(\beta_0, \boldsymbol{\theta})^{(s)}(\beta_0, \boldsymbol{\theta})^{(s)'} - (\beta_0, \boldsymbol{\theta})^*(\beta_0, \boldsymbol{\theta})^{*'}\}\right]\Big)$.

1.2 Move a Knot

In this step, if $D > 0$, a location change of one knot is proposed in a Metropolis-Hastings step. First a knot, l_{move} in $\boldsymbol{l}^{(s+1)} = \{l_1^{(s+1)}, \dots, l_{D^{(s+1)}+1}^{(s+1)}\}$ is randomly chosen to be moved with probability $\frac{1}{D^{(s+1)}+1}$. Since moves far away from the current location are unlikely to be accepted, a new knot location is drawn uniformly from unused candidates within some nearby window, $(l_{move} - w, l_{move} + w)$, to form a new proposed set of knots, $\boldsymbol{l}^*$. w can be used as a tuning parameter to alter the acceptance rate of moves. As above, $\boldsymbol{X}^*$ must be calculated for the proposed knots. The acceptance probability, $a(\boldsymbol{l}^*, \boldsymbol{l}^{(s+1)})$ =min(1, LAP) is then calculated. In this case, since all sets of knots of size $D^{(s+1)} + 1$ have the same prior probability, the ratio of the priors is 1. The proposal ratio depends on the number of vacant knots in the window for l_{move} and l^*, since all knots have the same probability of being moved and all potential locations in the window have the same probability of entering the model. The acceptance probability is

$$a(\boldsymbol{l}^*, \boldsymbol{l}^{(s+1)}) = \min\left(1, \frac{(\text{No. vacant in } (l_{move} - w, l_{move} + w))f(\boldsymbol{y}|\boldsymbol{l}^*, \dots)}{(\text{No. vacant in } (l^* - w, l^* + w)f(\boldsymbol{y}|\boldsymbol{l}^{(s+1)}, \dots)}\right)$$

Again, γ is then sampled from a $U(0, 1)$ distribution. If $a(\boldsymbol{l}^*, \boldsymbol{l}^{(s+1)}) > \gamma$, the knot is moved to the proposed location, and $\boldsymbol{l}^{(s+1)}$ is set to $\boldsymbol{l}^*$. Otherwise, $\boldsymbol{l}^{(s+1)}$

remains unchanged.

1.3 Update Fixed Effect Coefficients

The update of the fixed effects is separated into 2 blocks - the intercept and coefficients for the dropout-varying slope and the coefficients for any covariates included in the model. We use a Metropolis-Hastings step with a WLS proposal, as this method readily generalizes to non-normal outcomes in the exponential family and easily accommodates dimensional changes. To update $(\beta_0^{(s)}, \boldsymbol{\theta}^{(s)})'$, first calculate $\tilde{\boldsymbol{y}}_i = \boldsymbol{\eta}_i^{(s)} + (\boldsymbol{y}_i - \boldsymbol{\mu}_i^{(s)})g'(\boldsymbol{\mu}_i^{(s)}) - \boldsymbol{Z}_i\boldsymbol{\alpha}_i^{(s)} - \boldsymbol{C}_i\boldsymbol{\beta}_C^{(s)}$ and $\boldsymbol{W}_i^{-1} = b''(\boldsymbol{\eta}_i^{(s)})[g'(\boldsymbol{\mu}_i^{(s)})]^2$. The proposal distribution for the coefficients is $MVN(M, Cov)$, with $M = (\boldsymbol{R}_0^{-1} + \boldsymbol{X}'\boldsymbol{W}\boldsymbol{X})^{-1}(\boldsymbol{X}'\boldsymbol{W}\tilde{\boldsymbol{y}})$ and $Cov = (\boldsymbol{R}_0^{-1} + \boldsymbol{X}'\boldsymbol{W}\boldsymbol{X})^{-1}$, where $\boldsymbol{R}_0$ is the covariance matrix of the multivariate normal prior for the coefficients. β_0^* and $\boldsymbol{\theta}^*$ are drawn from the proposal distribution and the acceptance probability is calculated as:

$$a\{(\beta_0^*, \boldsymbol{\theta}^*), (\beta_0^{(s+1)}, \boldsymbol{\theta}^{(s+1)})\} = \frac{p(\boldsymbol{y}|\beta_0^*, \boldsymbol{\theta}^*, \dots)p(\beta_0^*, \boldsymbol{\theta}^*)q(\beta_0^{(s)}, \theta^{(s)})}{p(\boldsymbol{y}|\beta_0^{(s)}, \boldsymbol{\theta}^{(s)}, \dots)p(\beta_0^{(s)}, \boldsymbol{\theta}^{(s)})q(\beta_0^*\boldsymbol{\theta}^*)}$$

where $p(\beta_0, \theta)$ is the pdf of the multivariate normal prior for β_0 and θ and q is the pdf of the multivariate normal proposal distribution with mean M and variance Cov. γ is then sampled from a $U(0,1)$ distribution. If $a > \gamma$, then $\beta_0^{(s+1)} = \beta_0^*$ and $\boldsymbol{\theta}^{(s+1)} = \boldsymbol{\theta}^*$. Else $\beta_0^{(s+1)}$ and $\theta^{(s+1)}$ remain unchanged.

The fixed effects for the covariates can then be similarly updated by calculating $\tilde{\boldsymbol{y}}_i = \boldsymbol{\eta}_i^{(s)} + (\boldsymbol{y}_i - \boldsymbol{\mu}_i^{(s)})g'(\boldsymbol{\mu}_i^{(s)}) - \boldsymbol{Z}_i\boldsymbol{\alpha}_i^{(s)} - \beta_0^{(s+1)} - \sum_{k=1}^{D^{(s+1)}+1} \theta_k^{(s+1)}\tilde{u}_{ik}\boldsymbol{t}_i$ and using a MVN proposal with mean, $(\boldsymbol{R}_0^{-1} + \boldsymbol{C}'\boldsymbol{W}\boldsymbol{C})^{-1}(\boldsymbol{C}'\boldsymbol{W}\tilde{\boldsymbol{y}})$ and variance $(\boldsymbol{R}_0^{-1} + \boldsymbol{C}'\boldsymbol{W}\boldsymbol{C})^{-1}$.

1.4 Update Random Effects

For non-normal outcomes in the exponential family, the random effects are updated using a random walk Metropolis-Hastings step.

In the special case of a normally distributed outcome, the full conditional distribution of the random effects for subject i is normal with variance $\left(\boldsymbol{\Sigma}_\alpha^{-1(s)} + \sigma_\epsilon^{-2(s)}\boldsymbol{Z}_i'\boldsymbol{Z}_i\right)^{-1}$ and mean $\left(\boldsymbol{\Sigma}_\alpha^{-1(s)} + \sigma_\epsilon^{-2(s)}\boldsymbol{Z}_i'\boldsymbol{Z}_i\right)^{-1}\boldsymbol{Z}_i'\tilde{\boldsymbol{y}}_i\sigma_\epsilon^{-2(s)}$, where $\tilde{\boldsymbol{y}}_i = \boldsymbol{y}_i - \beta_0^{(s+1)} - \sum_{k=1}^{D^{(s+1)}+1} \theta_k^{(s+1)}\tilde{u}_{ik}\boldsymbol{t}_i - \boldsymbol{C}_i\boldsymbol{\beta}_C^{(s+1)}$. The random effects could be updated jointly, using a Gibbs step, however, it may be computationally more efficient to update the random intercept and random slope separately. The conditional distribution of the random intercept for subject i is normal with variance $\left\{\frac{n_i}{\sigma_\epsilon^{2(s)}} + \frac{1}{(1-\rho^2)\sigma_0^{2(s)}}\right\}^{-1}$ and mean

$\left\{\frac{n_i}{\sigma_\epsilon^{2(s)}} + \frac{1}{(1-\rho^2)\sigma_0^{2(s)}}\right\}^{-1} \left\{\frac{\sum_{j=1}^{n_i} \tilde{y_{ij}}}{\sigma_\epsilon^{2(s)}} + \frac{\rho\alpha_{1i}^{(s)}}{\sigma_0^{(s)}\sigma_1^{(s)}(1-\rho^2)}\right\}$,
where $\tilde{y}_{ij} = y_{ij} - \beta_0^{(s+1)} - \sum_{k=1}^{D^{(s+1)}+1} \theta_k^{(s+1)}\tilde{u}_{ik}t_{ij} - \boldsymbol{C}_{ij}\boldsymbol{\beta}_C^{(s+1)} - \alpha_{1i}^{(s)}t_{ij}$ and $\rho = \frac{\sigma_{01}^{(s)}}{\sigma_0^{(s)}\sigma_1^{(s)}}$.

Similarly, for subject i the conditional distribution of the random slope given the random intercept is also normal with variance $\left\{\frac{\sum_{j=1}^{n_i} t_{ij}^2}{\sigma_\epsilon^{2(s)}} + \frac{1}{(1-\rho^2)\sigma_1^{2(s)}}\right\}^{-1}$ and mean
$\left\{\frac{\sum_{j=1}^{n_i} t_{ij}^2}{\sigma_\epsilon^{2(s)}} + \frac{1}{(1-\rho^2)\sigma_1^{2(s)}}\right\}^{-1} \left\{\frac{\sum_{j=1}^{n_i} t_{ij}\tilde{y_{ij}}}{\sigma_\epsilon^{2(s)}} + \frac{\rho\alpha_{0i}^{(s)}}{\sigma_0^{(s)}\sigma_1^{(s)}(1-\rho^2)}\right\}$, where
$\tilde{y}_{ij} = y_{ij} - \beta_0^{(s+1)} - \sum_{k=1}^{D^{(s+1)}+1} \theta_k^{(s+1)}\tilde{u}_{ik}t_{ij} - \boldsymbol{C}_i\boldsymbol{\beta}_C^{(s+1)} - \alpha_{0i}^{(s+1)}$.

1.5 Update Variance Components

$\boldsymbol{\Sigma}_\alpha$ can be updated by sampling from the full conditional, which is distributed inverse Wishart $(\nu_0 + m, \boldsymbol{S} + \sum \boldsymbol{\alpha}_i'\boldsymbol{\alpha}_i)$, in a Gibbs step.

$$p(\boldsymbol{\Sigma}_\alpha|\boldsymbol{y},\dots) \propto \prod_{i=1}^{m} p(\boldsymbol{\alpha}_i|\boldsymbol{\Sigma}_\alpha)p(\boldsymbol{\Sigma}_\alpha)$$
$$\propto |\boldsymbol{\Sigma}_\alpha|^{-(n+\nu_0)/2} \exp\left[-0.5\mathrm{tr}\left\{\boldsymbol{\Sigma}_\alpha^{-1}\left(\boldsymbol{S} + \sum_{i=1}^{m}\boldsymbol{\alpha}_i'\boldsymbol{\alpha}_i\right)\right\}\right]$$

For a normally distributed outcome, the variance of the residual error, σ_ϵ^2, can also be updated in a Gibbs step by sampling from the full conditional inverse gamma distribution with parameters $a_0 + \sum_{i=1}^m n_i/2$ and $b_0 + \sum_{i=1}^m \sum_{j=1}^{n_i} \tilde{y}_{ij}^2/2$.

$$p(\sigma_\epsilon^2|\boldsymbol{y},\dots) \propto \prod_{i=1}^{m} p(\boldsymbol{y}_i|\boldsymbol{X}_i, \boldsymbol{Z}_i, \beta_0, \boldsymbol{\theta}, \boldsymbol{\beta}_C, \boldsymbol{\alpha}_i, \sigma_\epsilon^2, \boldsymbol{l})p(\sigma_\epsilon^2)$$
$$\propto \prod_{i=1}^{m}(2\pi\sigma_\epsilon^2)^{-n_i/2}\exp\left(-\sum_{j=1}^{n_i}\tilde{y}_{ij}^2/2\sigma_\epsilon^2\right)(\sigma_\epsilon^2)^{-a_0-1}\exp(-b_0/\sigma_\epsilon^2)$$

where $\tilde{y}_{ij} = y_{ij} - \beta_0^{(s+1)} - \sum_{k=1}^{D^{(s+1)}+1} \theta_k^{(s+1)}\tilde{u}_{ik}t_{ij} - \boldsymbol{C}_i\boldsymbol{\beta}_C^{(s+1)} - \boldsymbol{Z}_{ij}\boldsymbol{\alpha}_i^{(s+1)}$

1.6 Implementation Issues

In the dimension change step, the B-spline basis functions must be recalculated and a coefficient must be added or deleted. Formulas exist for updating coefficients when knots are added or deleted from a spline;[Lyche and Stom, 1996] however, using deterministic rules, the required symmetry of birth and death steps in RJMCMC is destroyed,[Biller, 2000] so a different method of updating coefficients is needed. In a birth step, Biller[Biller, 2000] has proposed adding

an additional coefficient by taking a weighted average of neighboring coefficients based on a random uniform number and adjusting the other coefficients accordingly. Applying this method to the BNSV to account for dropout results in low acceptance ratios, unless a large mean is used for the prior for the number of knots, as the proposed coefficients often fit the data poorly. Similarly, proposing a new coefficient from the prior distribution can also result in low acceptance of birth and death moves. Our approach to adding a coefficient described in Section 1 of the supplementary material is based on the WLS estimates of the coefficients when $\boldsymbol{l}^{(s)}$, the current set of knot locations, and $\boldsymbol{l}^{*}$, the proposed set of knot locations, are used for the natural cubic B spline transformation of dropout times and results in better mixing and higher acceptance rates (≈1-2%) in our dropout applications.

The method is not sensitive to starting values for the number or location of spline knots or for the coefficients, as Gammeran's WLS proposal typically has high acceptance rates (>70%) and coefficients are quickly updated. However, several factors do impact the number of knots included in the model, as well as how often births and deaths of knots are accepted. In addition to the prior mean for D, the values of b and d, σ^2_β and the variance of the proposal distribution when a new coefficient is added to the model all influence the acceptance probability of birth and death steps. In particular, choosing σ^2_β to be very large in order to be "uninformative" can result in low acceptance of birth moves and models with few or no knots; on the other hand, constricting the prior for the coefficients to a small range around 0 can result in the addition of many small coefficients into the model. For our problems, coefficients are typically small (absolute value less than 4) and σ^2_β of 25 to 100 appear strike a good balance between these two extremes. In our application, we favor smoothness and parsimony and typically choose a relatively small mean of 5 for D. We use b, d, and the variance of the proposal distribution when a new coefficient is added to the model to tune the acceptance probability of birth and death steps. We assess the convergence the MCMC chain by trace plots of the dropout time specific and marginal slope, as well as for the variance, intercept and covariate effects that do not involve varying numbers of spline components.

2 Additional Simulation Studies

Additional simulations were carried out as described in Section 3.1 of the main text, using linear dropout-varying slopes and no effect of dropout (Table 1). Results are shown in Table 2 and Figure 1. For the linear dropout effect, the BNSV has lower bias and MSE for the marginal slope than tradional GLMM's. For no dropout effect. the BNSV is able to fit the slope quite accurately, although the method has higher variance and MSE than the GLMM.

Table 1: Additional Simulation Study Comparing the BNSV and GLMM Methods: Linear and Constant Dropout Varying Slopes

Distribution	Form	$\beta_1(u)$
Normal	(iii)	$u-1$
Normal	(iv)	0.2
Binary	(iii)	$15u$
Binary	(iv)	7

Table 2: Additional Simulation Study Comparing the BNSV and GLMM Methods: Posterior Mean Estimates, Bias, Variance and Mean Squared Error for the Marginal Slope

		BNSV				GLMM			
Distribution	Form	Estimated Slope	Bias	Variance	MSE	Estimated Slope	Bias	Variance	MSE
Normal	(iii)	-0.496	**0.004**	0.004	**0.004**	-0.264	0.236	**0.0009**	0.057
Normal	(iv)	-0.199	0.001	0.001	0.001	-0.200	**0.0004**	**0.0007**	**0.0007**
Binary	(iii)	9.01	**0.01**	0.16	**0.16**	10.72	1.72	**0.13**	3.08
Binary	(iv)	7.05	0.05	0.06	0.06	7.05	0.05	**0.05**	0.06

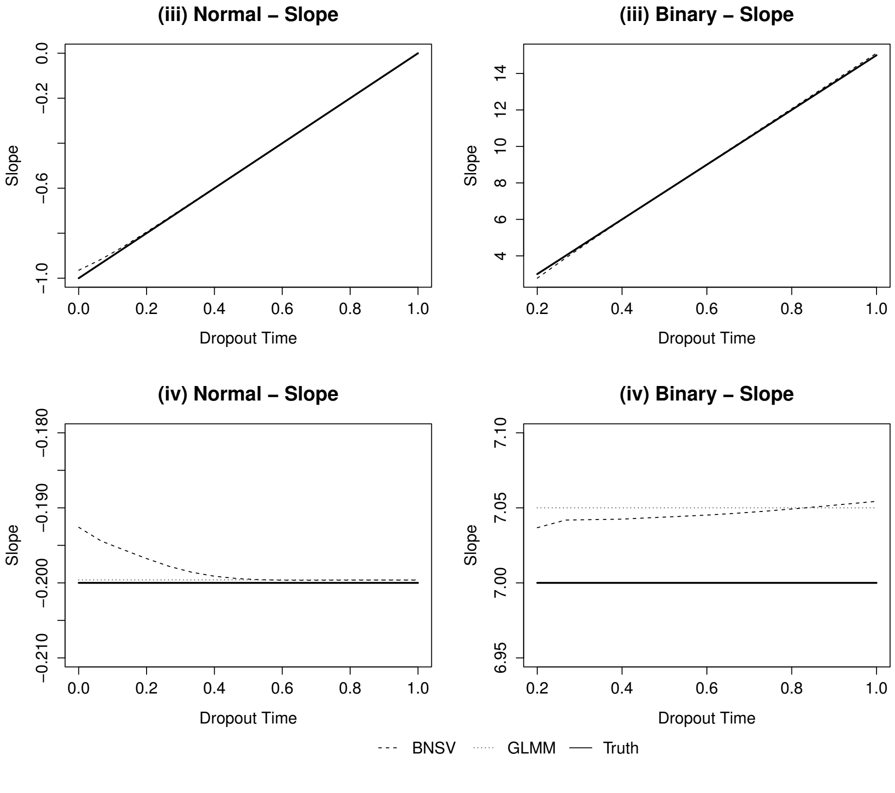

Figure 1: Additional Simulation Study of the BNSV: Dropout Varying Slopes

3 Sensitivity Analyses

We test the sensitivity of the results of the WIHS analyses to the assumption that subjects continue on the same linear trajectory after dropping out by considering a proportional attenuation of the slope after a subject's drop out, such that after dropping out, a subject's slope becomes $\delta\beta_1(u_i)$.

3.1 $CD4^+$ Decline

Estimates of $\ln(CD4^+)$ over time and plots of $CD4^+$ over time for hard drug users and others, again assuming a baseline $CD4^+$ count of 478.5, are presented in Table 3 and Figure 2 for $\delta = 0.75, 0.5, 0.25, 0$. A flat slope after dropping out ($\delta = 0$, similar to last observation carried forward) is unrealistic, as $CD4^+$ count is known to decline over time. Differences between drug users and others are reduced but remain statistically significant for $\delta = 0.75, 0.5, 0.25$, indicating our results are robust to reasonable violations of the assumption that subjects maintain the same decline in $CD4^+$ after dropout.

3.2 Viral Load Suppression

Estimates of ln(odds of suppression) over time and plots of the probability of viral load suppression over time for recreational drug users and others, again assuming a baseline $CD4^+$ count of 267 and $\log_{10}$(viral load) of 4.2, are presented in Table 4 and Figure 3 for $\delta = 0.75, 0.5, 0.25, 0$. Drug users have lower odds of suppression compared to other subjects that initiated HAART under all of the sensitivity analysis scenarios. As in the primary analysis, these differences are not statistically significant.

Table 3: Sensitivity Analyses: Estimated Ln($CD4^+$) for Untreated Subjects in the WIHS for a Range of δ. PM=posterior mean, CI=credible interval, PP=posterior probability of a difference > 0/others having higher $CD4^+$ than hard drug users.

δ	Year	Non-User			Drug User			Difference			PP
		Mean	95% CI		Mean	95% CI		Mean	95% CI		
0.75	1	6.025	5.986	6.063	5.864	5.792	5.936	0.161	0.076	0.246	1.000
	2	5.852	5.793	5.910	5.532	5.387	5.676	0.320	0.164	0.477	1.000
	3	5.692	5.608	5.773	5.211	4.981	5.440	0.480	0.238	0.724	1.000
	4	5.535	5.426	5.641	4.895	4.577	5.211	0.640	0.310	0.973	1.000
0.5	1	6.028	5.989	6.066	5.889	5.823	5.956	0.139	0.058	0.219	1.000
	2	5.878	5.823	5.932	5.634	5.521	5.746	0.244	0.117	0.372	1.000
	3	5.754	5.681	5.825	5.400	5.232	5.568	0.354	0.171	0.538	1.000
	4	5.637	5.546	5.725	5.176	4.949	5.401	0.461	0.220	0.705	1.000
0.25	1	6.031	5.993	6.069	5.915	5.852	5.978	0.116	0.038	0.194	0.998
	2	5.904	5.853	5.955	5.735	5.650	5.821	0.169	0.065	0.273	0.999
	3	5.816	5.753	5.878	5.589	5.476	5.702	0.227	0.095	0.360	1.000
	4	5.738	5.663	5.812	5.456	5.314	5.598	0.282	0.120	0.446	1.000
0	1	6.034	5.996	6.072	5.941	5.880	6.002	0.093	0.017	0.170	0.992
	2	5.931	5.883	5.978	5.837	5.767	5.907	0.093	0.002	0.185	0.978
	3	5.878	5.823	5.933	5.778	5.699	5.856	0.100	-0.002	0.204	0.973
	4	5.839	5.777	5.902	5.737	5.650	5.822	0.103	-0.010	0.218	0.963

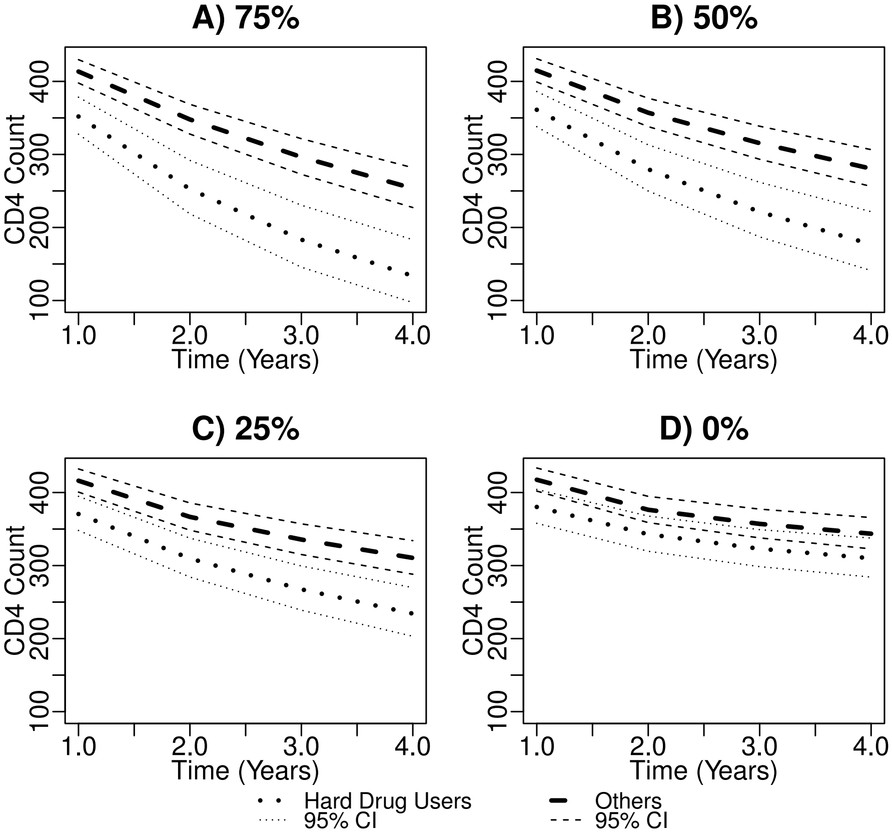

Figure 2: Sensitivity Analysis: Estimated CD4$^+$ Over Time in Untreated Subjects in the WIHS Assuming a Proportional Attenuation of the Slope after Dropout

Table 4: Sensitivity Analyses: Estimated Ln(Odds of Suppression) for Treated Subjects in the WIHS for a Range of δ. PM=posterior mean, CI=credible interval, PP=posterior probability of a difference $>$ 0/others having higher odds of suppression than recreational drug users.

δ	Year	Non-User			Drug User			Difference			PP
		Mean	95% CI		Mean	95% CI		Mean	95% CI		
0.75	2	−0.941	−1.130	−0.747	−1.059	−1.429	−0.700	0.118	−0.285	0.528	0.72
	4	−0.682	−0.884	−0.481	−0.894	−1.356	−0.478	0.212	−0.255	0.710	0.82
	6	−0.417	−0.664	−0.177	−0.726	−1.353	−0.190	0.309	−0.289	0.980	0.85
	8	−0.149	−0.462	0.150	−0.559	−1.385	0.124	0.409	−0.351	1.281	0.86
	10	0.115	−0.270	0.486	−0.394	−1.424	0.450	0.509	−0.428	1.596	0.86
0.50	2	−0.928	−1.115	−0.734	−1.044	−1.405	−0.689	0.117	−0.279	0.517	0.72
	4	−0.642	−0.833	−0.448	−0.855	−1.258	−0.473	0.213	−0.215	0.655	0.84
	6	−0.343	−0.566	−0.125	−0.659	−1.164	−0.202	0.316	−0.196	0.866	0.89
	8	−0.040	−0.310	0.220	−0.466	−1.097	0.088	0.426	−0.195	1.107	0.92
	10	0.257	−0.067	0.570	−0.278	−1.049	0.386	0.535	−0.204	1.360	0.93
0.25	2	−0.914	−1.100	−0.721	−1.029	−1.382	−0.679	0.115	−0.272	0.509	0.72
	4	−0.601	−0.783	−0.415	−0.816	−1.173	−0.463	0.214	−0.180	0.612	0.86
	6	−0.269	−0.469	−0.069	−0.593	−0.993	−0.203	0.324	−0.113	0.771	0.93
	8	0.069	−0.162	0.296	−0.374	−0.837	0.068	0.443	−0.053	0.960	0.96
	10	0.399	0.129	0.663	−0.162	−0.699	0.340	0.562	−0.007	1.160	0.97
0.00	2	−0.901	−1.086	−0.708	−1.014	−1.363	−0.672	0.114	−0.266	0.502	0.72
	4	−0.561	−0.735	−0.379	−0.776	−1.105	−0.449	0.215	−0.149	0.586	0.88
	6	−0.195	−0.376	−0.008	−0.526	−0.861	−0.192	0.331	−0.044	0.716	0.96
	8	0.179	−0.023	0.383	−0.282	−0.637	0.073	0.461	0.060	0.870	0.99
	10	0.541	0.313	0.772	−0.046	−0.434	0.337	0.588	0.151	1.036	1.00

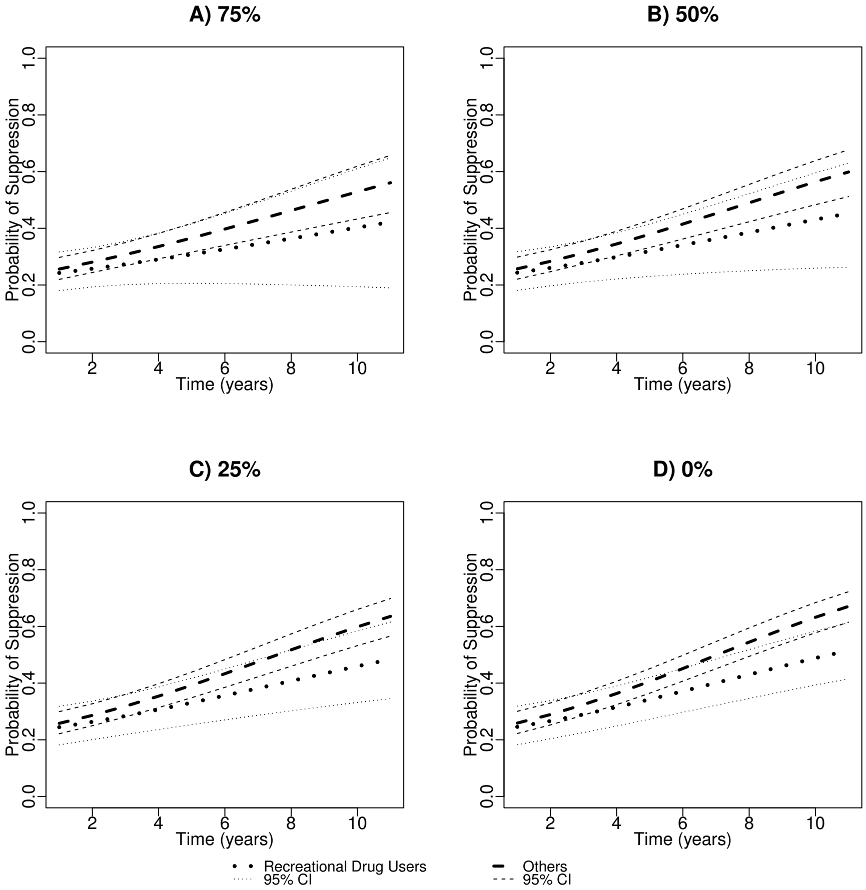

Figure 3: Sensitivity Analysis: Estimated Probability of Viral Load Suppression Over Time for Treated Subjects in the WIHS Assuming a Proportional Attenuation of the Slope after Dropout

4 Computing Times

Table 5: Times (minutes) to complete the analysis of a single dataset from the normal and binary outcome simulation studies and the WIHS analyses of $CD4^+$ T cell count and viral load suppression using a MacBook Pro with 3.5 GHz Intel Core i7 processor and 16 GB of RAM.

				Time (minutes)		
Dataset	MCMC Iterations	N Subjects	N Observations	BNSV	CLM	Frailty
Normal (i)	40,000	400	3,359	8.8	2.7	2.2
Binary (i)	40,000	400	4,048	19.1	4.7	4.3
WIHS $CD4^+$	200,000	814	3,196	72.5	40.9	22.2
WIHS viral load suppression	200,000	1,015	15,909	372.0	280.5	198.2

References

C. Biller. Bayesian Regression Splines in Semiparametric Generalized Linear Models. *Journal of Computational and Graphical Statistics*, 9(1):122–140, 2000.

T. Lyche and K. Stom. Knot insertion for natural splines. *Annals of Numerical Mathematics*, 3:221–246, 1996.
